# Oxidative Synthesis of Acid Blue 7 Dye Catalyzed by CuO/Silicotungstic Acid in Water-Phase

**DOI:** 10.3390/ma14164505

**Published:** 2021-08-11

**Authors:** Akihiro Nomoto, Tomoya Okada, Yuki Yamamoto, Shota Kuroda, Kuniaki Marui, Mika Yamamoto, Hidetaka Tsujimoto, Michio Ueshima, Tamotsu Nishigahana, Keiji Itoh, Gohei Kobata, Shintaro Kodama, Akiya Ogawa

**Affiliations:** 1 Department of Applied Chemistry, Graduate School of Engineering, Osaka Prefecture University, 1-1 Gakuen-cho, Nakaku, Sakai, Osaka 599-8531, Japan; 40ogntks@gmail.com (T.O.); syb02137@edu.osakafu-u.ac.jp (Y.Y.); sab02061@edu.osakafu-u.ac.jp (S.K.); kuniaki.marui@honshuchemical.co.jp (K.M.); yamamotomiike620@gmail.com (M.Y.); ueshima.m@zeus.eonet.ne.jp (M.U.); skodama@chem.osakafu-u.ac.jp (S.K.); ogawa@chem.osakafu-u.ac.jp (A.O.); 2 Environmental and Materials Chemistry, Department of Technological Systems, Osaka Prefecture University College of Technology, 26-12 Saiwai-cho, Neyagawa-shi, Osaka 572-8572, Japan; h-tsujimoto@osaka-pct.ac.jp; 3 Kobata Sangyo Co., Ltd., 1-6-22 Kyomachibori, Nishi-ku, Osaka 550-0003, Japan; ta-nishigahana@kbts.jp (T.N.); kei_itoh2911@zeus.eonet.ne.jp (K.I.); go-kobata@kbts.jp (G.K.)

**Keywords:** triarylmethane, acid blue 7, catalytic oxidation, silicotungstic acid

## Abstract

A catalytic oxidation reaction for Acid Blue 7 dye synthesis was evaluated in water. Without lead oxide or manganese oxide derivatives as oxidants, polyoxometalate catalysts were investigated to reduce the usage of harmful heavy metal. A catalyst was prepared by mixing silicotungstic acid with copper oxide, and aqueous hydrogen peroxide (30%) was used as an oxidizing agent. This reaction proceeded to produce Acid Blue 7 from the corresponding leuco acid after 45 min at 95 °C and was viable for a 10 g-scale synthesis.

## 1. Introduction

In the synthesis of dyes, oxidation is one of the most important processes for organic synthesis and industrial chemistry. Interest in triarylmethane dyes, such as crystal violet and malachite green, has grown in recent years [1,2,3]. In addition to conventional dyeing applications, the demand for color filters for liquid crystal displays or bio-probes has increased substantially [4,5,6,7].

**Scheme 1:**
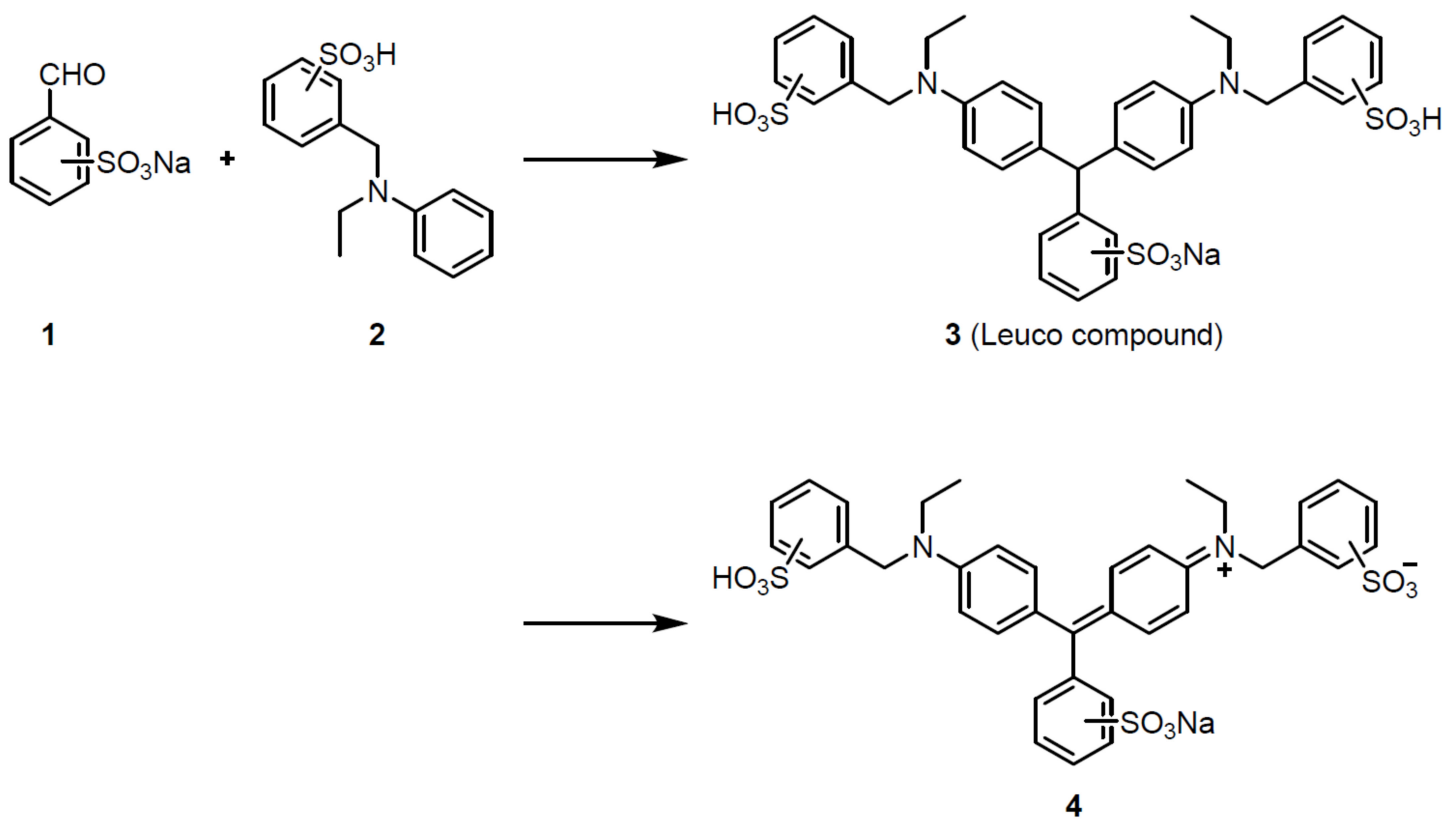
The synthetic route for triarylmethane dye.

As shown in Scheme 1, triarylmethane dyes (4) are produced by condensing benzaldehydes (1) with *N*-ethyl-*N*-phenylbenzylamine (2) to form the corresponding leuco compounds (3), followed by oxidation with dichromate, lead peroxide, or manganese oxide in water. [8,9,10,11,12,13,14,15]. Fluorinated analogs of malachite green were synthesized and their toxicity was investigated. The toxicity of malachite green was reduced by fluorination. In this synthesis, 0.5 mmol of the corresponding leuco acid was oxidized by 0.65 mmol of PbO_2_ [16]. The oxidation reactivity was also studied [17]. Comparing K_2_S_2_O_8_, KMnO_4_, and MnO_2_, MnO_2_ was the best oxidant for the synthesis of malachite green. In this report, while it contains sulfur, K_2_S_2_O_8_ seems to be an attractive candidate as an oxidant. Although it has been used industrially as an oxidant, it is highly harmful and affects the environment and human body. Accordingly, environmentally benign oxidation methods are required [18,19]. Acid Blue 9 has been previously synthesized from the corresponding leuco acid by clean oxidation using a metal-complex catalyst [19]. Accordingly, an iron phthalocyanine derivative catalyzed this oxidation reaction and diluted H_2_O_2_ (10%) was used as an oxidant. Although this catalyst ligand must be prepared from ferric phthalocyanine chloride in two steps, this reaction was viable for pilot scale production and, thus, these results are notable from a green chemistry perspective.

In our laboratory, we have previously developed an organocatalytic or metal-free oxidation method [20,21,22]. We reported on the synthesis of triarylmethanes from benzylamines via an organocatalytic oxidative C–N bond cleavage and subsequent double C–C bond formation, followed by conversion into a series of triarylmethane blue dyes by organic oxidants, such as chloranil or DDQ [23].

**Scheme 2:**
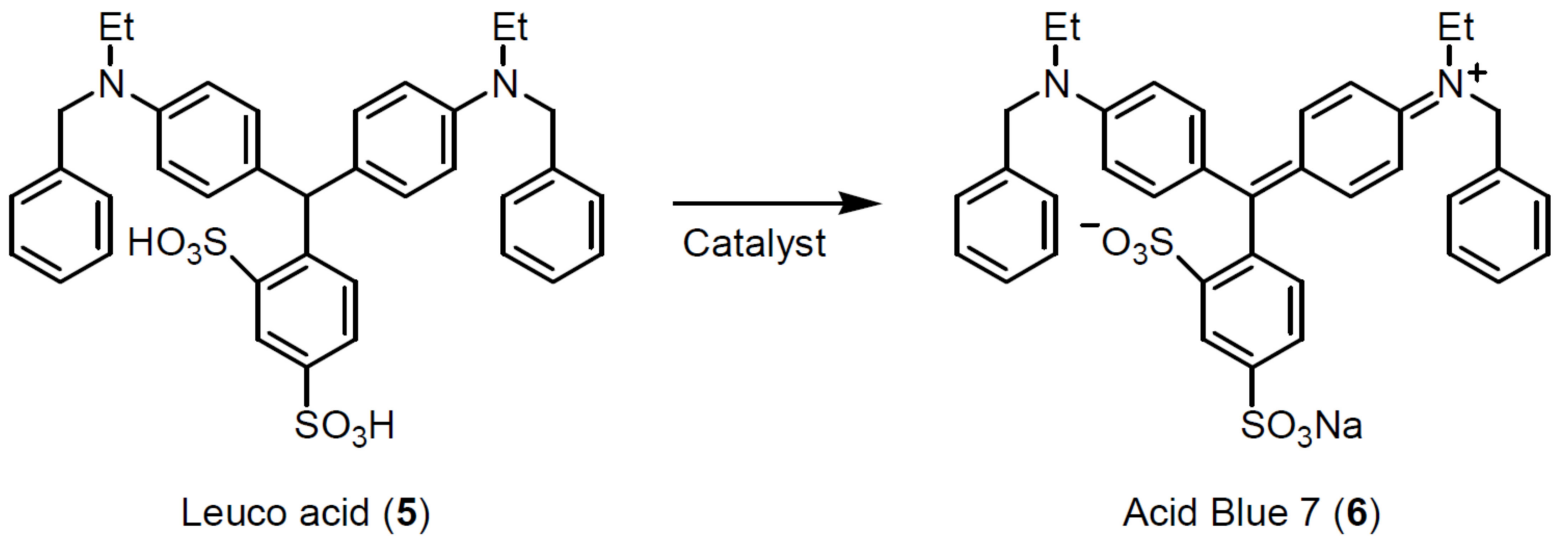
Schematic representation for Acid Blue 7 synthesis.

However, this system has been established only in the organic phase and is difficult to carry out in the water phase. Therefore, it is so limited to synthesize this type of dyes via leuco acids in spite of ease with which their derivatives are obtained. In this study, we thus established a novel catalytic oxidation reaction for the synthesis of triarylmethane blue dyes in water by using commercially available aqueous 30% hydrogen peroxide (H_2_O_2_). As shown in Scheme 2, we selected leuco acid (5) as a typical substrate and attempted to transform this compound into *N*-[4-[(2,4-disulfophenyl)[4-[ethyl(phenylmethyl)amino]phenyl]methylene]-2,5-cyclohexadien-1-ylidene]-*N*-ethyl-benzenemethanaminium sodium salt (Acid Blue 7) (6).

From this viewpoint, the work aimed to find an efficient catalytic oxidation using H_2_O_2_ as an oxidant for preparing Acid Blue 7 as a model compound, hopefully reducing the use of heavy metal oxidants. To establish this oxidation system, an accessible ligand should be used. Although we have previously studied vanadium-catalyzed oxidation with H_2_O_2_ or oxygen as an oxidant [24,25,26,27], these catalysts are very hard to prepare. Therefore, we used commercially available polyoxometalate catalysts coordinated with metal ions [28,29,30,31,32].

## 2. Materials and Methods

### 2.1. General and Material

^1^H-NMR spectra were recorded on a JEOL JNM-AL-400 (Tokyo, Japan) spectrometer using CD_3_OD as the solvent and tetramethylsilane as an internal standard. High resolution ESI mass spectra were measured on WATERS Synapt G2 (Waters Japan, Tokyo, Japan) instruments. All materials were obtained from commercial suppliers and were used without further purification.

High-performance liquid chromatography (HPLC) measurements were performed with an EXTREMA instrument (JKASCO, Tokyo, Japan). Leuco acid was obtained from Kobata Sangyo (Wakayama, Japan). Silicotungstic acid and other polyoxometalates were obtained from Japan New Metals (Osaka, Japan), and all other chemicals and solvents were purchased from FUJIFILM Wako Chemicals (Tokyo, Japan) and used without further purification. Acid Blue 7 (Alphazurine A) was purchased from Santa Cruz (Dallas, USA), and purified by COSMOSIL 140C_18_-OPN (Nacarai, Kyoto, Japan) for column chromatography with methanol as an HPLC standard. ^1^H-NMR (400 MHz) δ 8.61 (d, *J* = 1.4 Hz, 1H), 7.95 (dd, *J* = 7.8, 1.4 Hz, 1H), 7.41-7.21 (m, 14H), 7.16 (d, *J* = 7.8 Hz, 1H), 6.97 (d, *J* = 9.2 Hz, 4H), 4.86 (overlapped, 4H), 3.75 (td, *J* = 15.2, 7.5 Hz, 4H), 1.34-1.27 (m, 6H).

### 2.2. Oxidation Reaction on a Small Scale

The oxidation reaction was carried out as follows: briefly, to prepare the leuco solution, the leuco acid and sodium carbonate were stirred until dissolved in water at room temperature for half a day. Next, as a catalyst solution, metal ions (or metal oxides) and polyoxometalates were mixed by stirring in an ordinary manner. Initially, metal oxides were used, and the catalyst solution was suspended in water. After stirring for half a day, the catalyst solution was clearly dissolved. The catalyst solutions and leuco solutions were mixed in a two-necked flask as a starting mixture. The oxidation reaction was carried out by adding 30% H_2_O_2_ aq. dropwise over 5 min to this starting mixture at an appropriate temperature. The oxidation process was monitored by HPLC. HPLC measurements were carried out in the conventional manner (λ = 254 nm, eluent = CH₃CN:0.05% H₃PO₄ aq. = 7:3, 1 mL/min). Yields were calculated from the calibration curve of purified Acid Blue 7.

### 2.3. 10 G-Scale Synthesis

The oxidation reaction was almost the same in the small scale-synthesis. The leuco acid and sodium carbonate were dissolved in 250 mL of water and a catalyst solution was prepared in 250 mL of water. These solutions were mixed in a 1 L two-necked flask and the oxidation reaction was carried out by adding 30% H_2_O_2_ aq. in the same way. However, it took a longer time to add H_2_O_2_ aq. dropwise and 45 min was needed to complete the process. After 45 min, the reaction mixture was divided into small portions, poured into Petri plates and air-dried. At this point, 12.9 g of crude compounds was obtained containing undefined byproducts and water. A purified compound was obtained by reversed-phase column chromatography (Cosmosil 140C_18_-OPN) in 69%. M.p. 205 °C (decomp.); ^1^H-NMR (400 MHz) δ 8.63 (s, 1H), 7.95 (d, *J* = 6.9 Hz, 1H), 7.41-7.21 (m, 14H), 7.16 (d, *J* = 6.9 Hz, 1H), 6.97 (d, *J* = 8.7 Hz, 4H), 4.86 (overlapped, 4H), 3.77-3.70 (m, 4H), 1.29 (t, *J* = 3.7 Hz, 6H); ^13^C-NMR (100 MHz) δ 167.5, 164.5, 148.5, 132.8, 128.3, 123.8, 120.8, 119.5, 119.5, 118.8, 118.4, 117.8, 105.7, 45.9, 45.7, 38.7, 38.6, 3.5; ESI-HRMS (M/z) [M+] calcd for C_37_H_36_N_2_O_6_S_2_ 668.2015, found 668.1968.

## 3. Results

**Figure 1:**
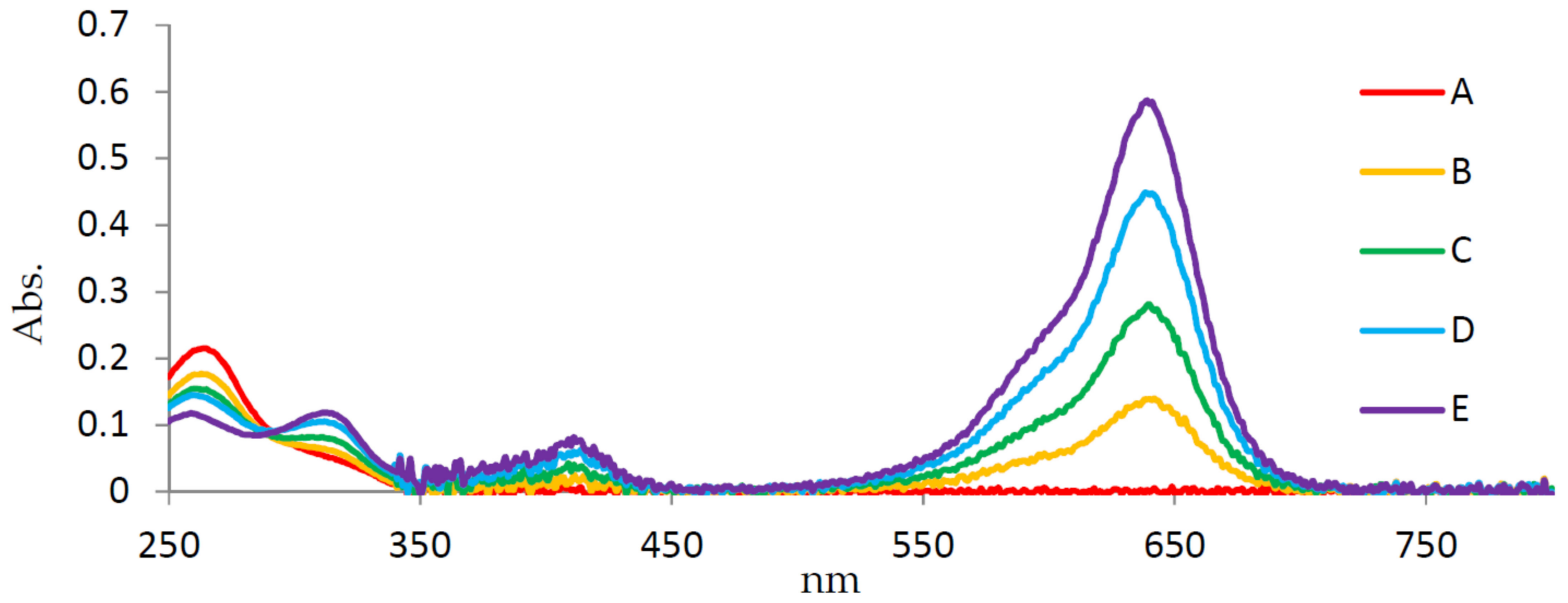
Ultraviolet–visible spectra of leuco acid (5), Acid Blue 7 (6), and mixtures. Concentrations of mixtures of leuco acid and Acid Blue 7 (leuco acid:Acid Blue 7, 10^−5^ M) are as follows: A = 1.0:0; B = 0.75:0.25; C = 0.5:0.5; D = 0.25:0.75; E = 0:1.0.

**Figure 2:**
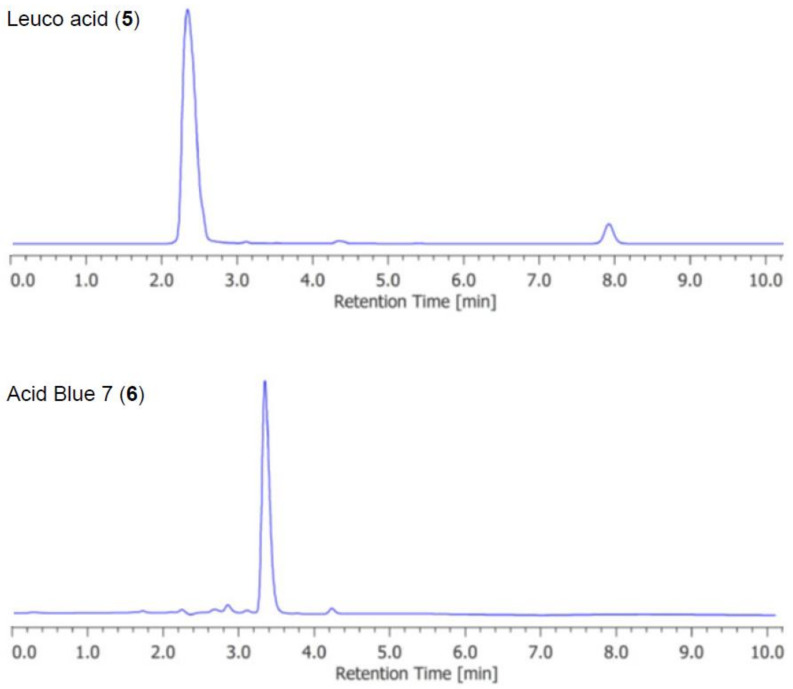
HPLC charts for leuco acid (**top**) and Acid Blue 7 (**bottom**) (λ = 254 nm, CH₃CN:0.05% H₃PO₄ aq. = 7:3).

First, we checked the spectra of both leuco acid and the desired product, Acid Blue 7. The total amount of substance was adjusted to 1.0 × 10^−5^ M, and each solution was measured by ultraviolet–visible spectroscopy. The spectra are shown in Figure 1. From these results, the desired Acid Blue 7 was detected easily; however, the disappearance of the starting leuco acid was difficult to detect. Thus, we next used HPLC measurements to monitor the oxidation process. By HPLC measurements (Figure 2), both leuco acid and Acid Blue 7 were easily identified.

**Scheme 3:**
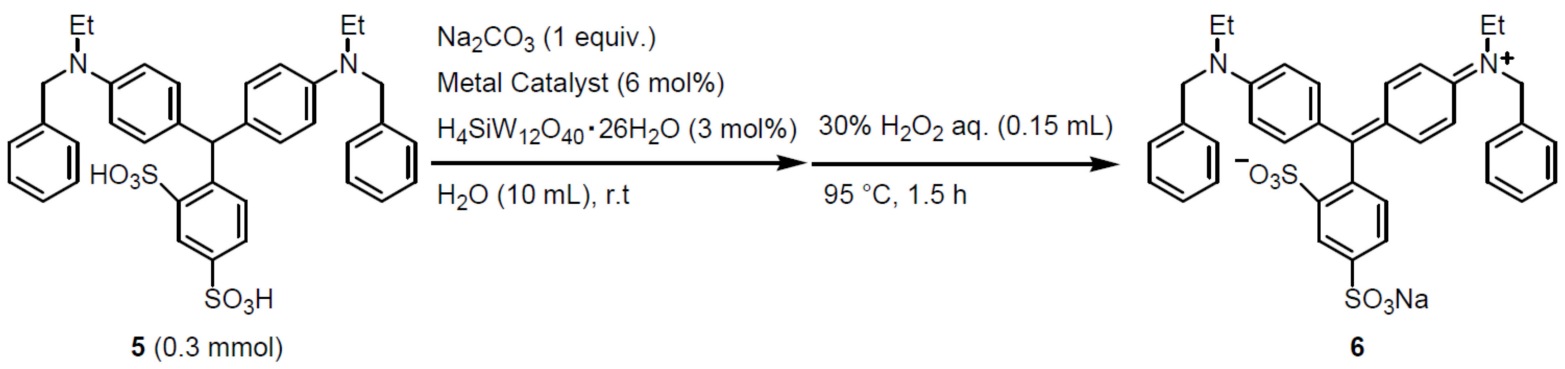
Oxidation catalyzed by various metal catalyst.

**Scheme 4:**
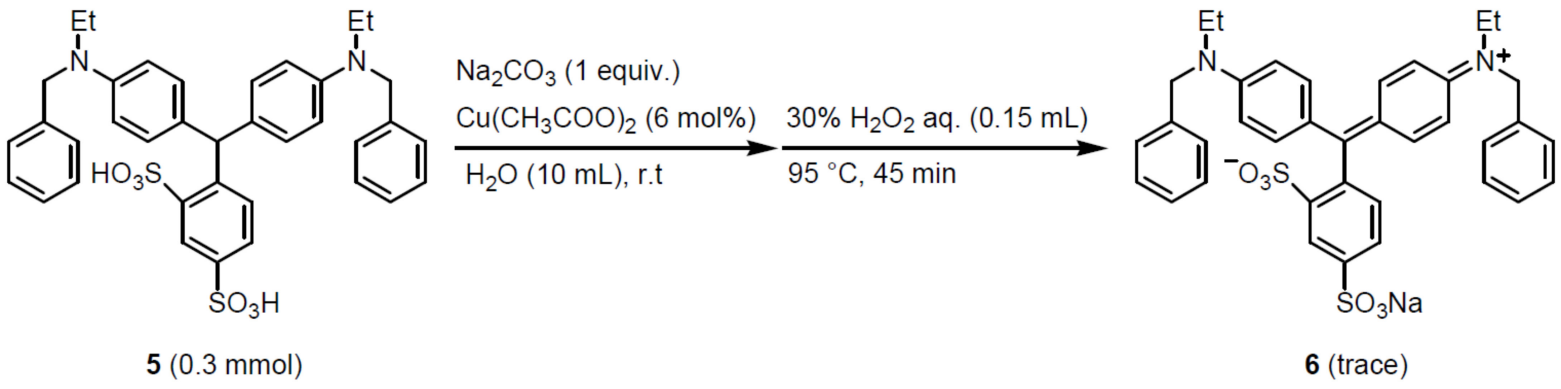
Oxidation with copper(II) acetate.

Catalyst screening was performed using several metal species with silicotungstic ac-id as a typical polyoxometalate for 1.5 h at 95 °C (Scheme 3), and the results are summarized in Table 1. Although some metal oxides failed to form a clear catalyst solution, iron and copper oxide were efficient catalysts (entries 7–11). In particular, when CuO was used as the metal catalyst, a 78% yield of Acid Blue 7 was obtained (entry 11). We also examined this oxidation with copper(II) acetate without silicotungstic acid as a water-soluble catalyst, but the desired product was not obtained (Scheme 4), and polyphosphoric acid, phosphotungstic acid, or phosphomolybdic acid were not effective for this oxidation. These results suggested that coordination with silicotungstic acid was essential for this oxidation catalyst.

**Scheme 5:**
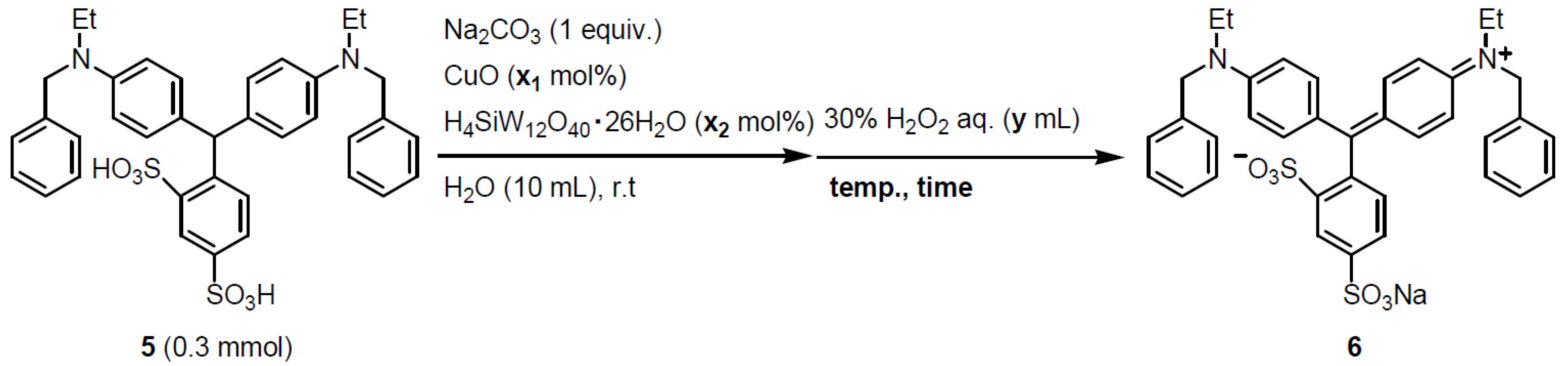
Representation for various amounts of CuO, silicotungstic acid, and H_2_O_2_.

Based on these results, the reaction conditions were optimized (Scheme 5). The oxidation conditions were achieved by changing the amounts of CuO (X_1_ mol%) and silicotungstic acid (X_2_ mol%) as well as the reaction temperature and reaction time (Table 2).

Even if the amount of catalyst and H_2_O_2_ solution was doubled, the yield did not change significantly (entries 1 and 2). The yield also decreased when the reaction temperature (in an oil bath) was lowered to 80 °C (entry 3), and the reaction mixture became a complex mixture under reflux conditions (entry 4). The reaction proceeded sufficiently evenly when the reaction time was shortened to 45 min (entry 5).

## 4. Discussion

Acid blue dyes have low heat resistance, so the elongation of the reaction time or an increase in the amount of H_2_O_2_ causes decomposition and leads to a decrease in the reaction yield. Contrarily, a lower reaction temperature also decreased the reaction yield. An appropriate reaction time and temperature are thus essential for this oxidation.

**Figure 3:**
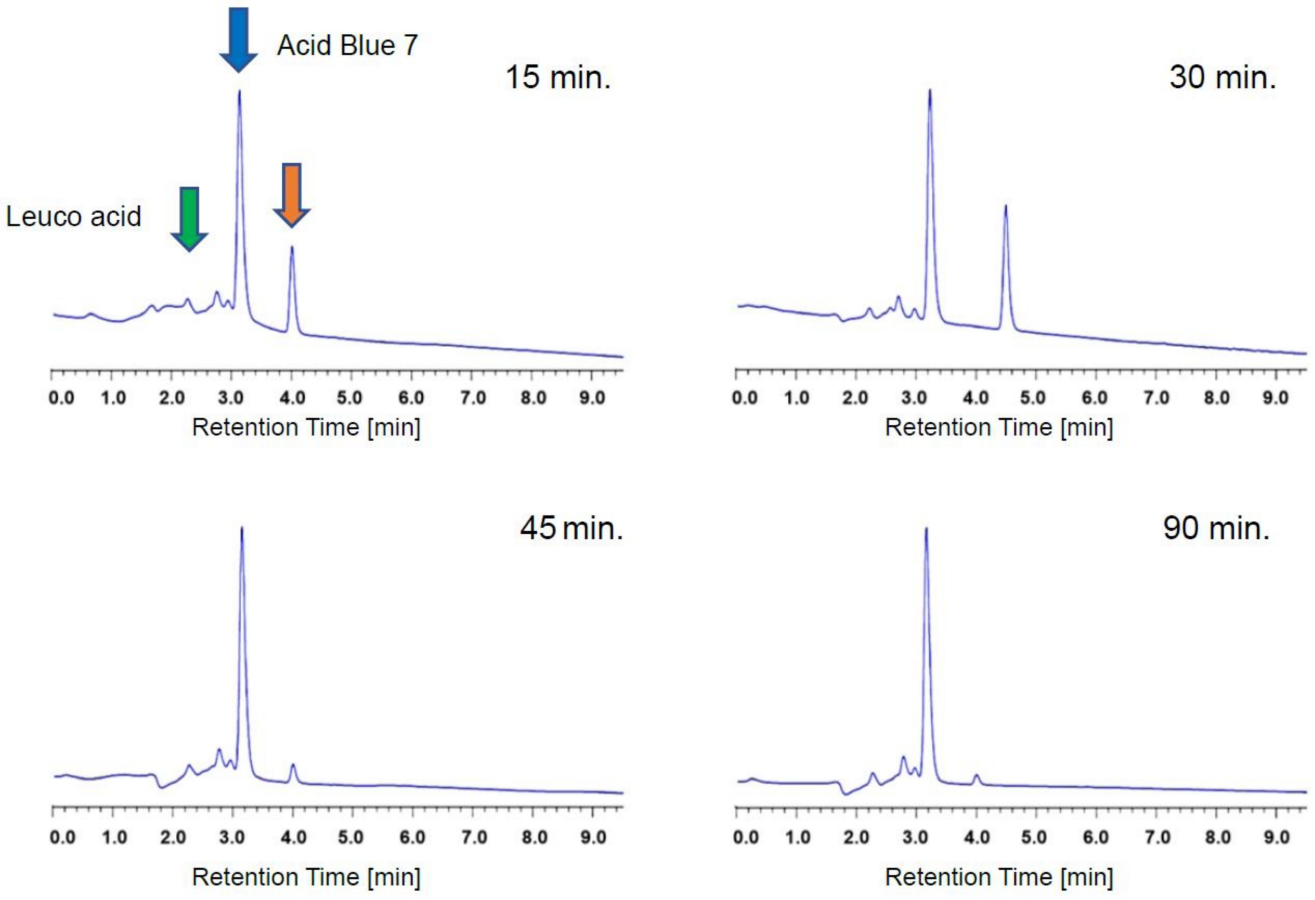
Time-course monitoring (15, 30, 45, and 90 min.) by HPLC.

We monitored this reaction using HPLC (Figure 3). In the early stage (15 min) of oxidation, the initial leuco acid disappeared, and another compound was detected after around 4 min (see the HPLC chart, orange arrow). As the reaction proceeded, this compound disappeared after 45 min.

**Scheme 6:**
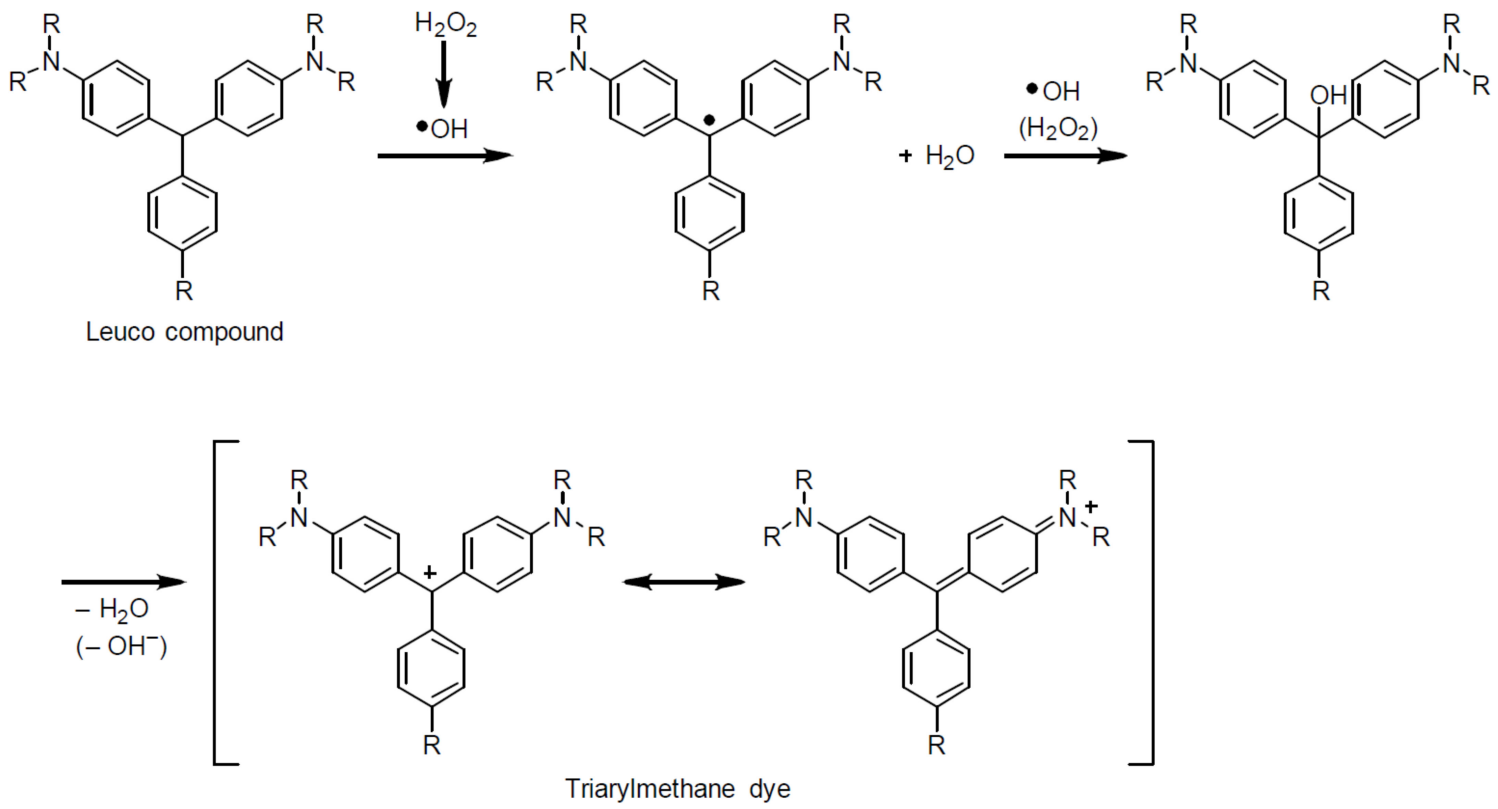
Reported plausible pathway for generation of triarylmethane dye.

According to the predicted reaction mechanisms [33,34,35,36,37] and the results of the present HPLC measurements, the oxidation reaction proceeded in two steps (Scheme 6).

The hydroxyl radical generated from H_2_O_2_ attacked the tertiary hydrogen of the leuco acid to form a tertiary radical and water. This tertiary radical reacted with hydrogen peroxide (15–30 min). Dehydroxylation then occurred to produce the desired product. We have not yet been able to detect the intermediate and the details of the reaction mechanism are under investigation.

This catalytic system was also applied to a 10 g-scale synthesis. A 15 mmol portion of leuco acid (10.07 g) was subjected to oxidation. After the addition of H_2_O_2_, while it takes 60 min by scale-up, the reaction mixture was stirred for more 45 min. After the reaction, the reaction mixture was divided into small portions, and poured into Petri plates, and air-dried. At this point, 12.9 g of crude compounds were obtained, containing undefined by products and water. Although small amounts of impurity were observed from HPLC measurement (Supplementary Materials, Figure S5), a purified compound was obtained by reversed-phase column chromatography (Cosmosil 140C_18_-OPN) in 69%.

## 5. Conclusions

The catalytic oxidation of triarylmethane can be used to form a blue dye. The coordination of silicotungstic acid with CuO has been found to be an efficient catalyst for water-phase oxidation. However, environmentally benign processes have recently attracted considerable attention from a sustainable-manufacturing perspective. In this report, Acid Blue 7 was prepared from the corresponding leuco acid with commercially available aqueous 30% H_2_O_2_ without toxic Cr or Pb species as oxidants. Although our ability to scale the reaction was limited by the use of common laboratory apparatus and it took a long time for addition of H_2_O_2_ dropwise, this reaction was demonstrated to be applicable to at least a 10 g-scale synthesis. This oxidation system may lead to the development of ecofriendly dye production methods in the future.

## Figures and Tables

**Table 1:** Catalyst screening.

Entry	Metal Catalyst	Yield (%) *^a^*
entry 1	BaSO_4_	n.d.
entry 2	MgI_2_	trace
entry 3	MgO	n.d.
entry 4	MgCl_2_	10
entry 5	MgCl_2_·6H_2_O	n.d.
entry 6	(CH_3_COO)_2_·4H_2_O	n.d.
entry 7	FeSO_4_·7H_2_O	30
entry 8	FeCl_3_·6H_2_O	13
entry 9	FeCl_2_	13
entry 10	FeO	12
entry 11	CuO	78

*^a^* Determined by HPLC (λ = 254 nm, CH₃CN:0.05% H₃PO₄ aq. = 7:3).

**Table 2:** Optimization of reaction conditions.

Entry	X_1_/X_2_	Y (mL)	Temp. (°C) *^c^*	Time (min)	Yield (%) *^a^*
entry 1	12/6	0.15	95	90	79
entry 2	6/3	0.30	95	90	62
entry 3	6/3	0.15	80	90	26
entry 4	6/3	0.15	110	90	64 *^b^*
entry 5	6/3	0.15	95	45	74

*^a^* Determined by HPLC (λ = 254 nm, CH₃CN:0.05% H₃PO₄ aq. = 7:3). *^b^* Under reflux condition. *^c^* Oil bath temperature.

## Data Availability

The data presented in this study are available on request from the corresponding author.

## Supplementary Materials

The following are available online at https://www.mdpi.com/article/10.3390/ma14164505/s1, Figure S1: 1H-NMR of purchased and purified Acid Blue 7 (Alphazurine A), Figure S2: 1H-NMR of synthesized Acid Blue 7, Figure S3: 13C-NMR of synthesized Acid Blue 7, Figure S4: Mass spectra of synthesized Acid Blue 7. Figure S5: HPLC results of 10 gram-scale synthesis; before purification (left) and after purification (right) by column chromatog-raphy.
