# Supplementary material for: Oxidative Synthesis of Acid Blue 7 Dye Catalyzed by CuO/Silicotungstic Acid in Water-Phase
Source: Materials (Basel). 2021 Aug 11;14(16):4505. doi: 10.3390/ma14164505 (PMC8400946; doi:10.3390/ma14164505)
Supplement: Supplementary file 1 [file materials-14-04505-s001.zip › materials-1298063-supplementary.pdf]

materials

MDPI

Supplementary Materials

Oxidative Synthesis of Acid Blue 7 Dye Catalyzed by CuO/Silicotungstic Acid in Water-Phase

Akihiro Nomoto [1,*], Tomoya Okada [1], Yuki Yamamoto [1], Shota Kuroda [1], Kuniaki Marui [1], Mika Yamamoto [1], Hidetaka Tsujimoto [2], Michio Ueshima [1], Tamotsu Nishigahana [3], Keiji Itoh [3], Gohei Kobata [3], Shintaro Kodama [1] and Akiya Ogawa [1]

[1] Department of Applied Chemistry, Graduate School of Engineering, Osaka Prefecture University, 1-1 Gakuen-cho, Nakaku, Sakai, Osaka 599-8531, Japan; 40ogntks@gmail.com (T.O.); syb02137@edu.osakafu-u.ac.jp (Y.Y.); sab02061@edu.osakafu-u.ac.jp (S.K.); kuniaki.marui@honshuchemical.co.jp (K.M.); yamamotomiike620@gmail.com (M.Y.); ueshima.m@zeus.eonet.ne.jp (M.U.); skodama@chem.osakafu-u.ac.jp (S.K.); ogawa@chem.osakafu-u.ac.jp (A.O.)

[2] Environmental and Materials Chemistry, Department of Technological Systems, Osaka Prefecture University College of Technology, 26-12 Saiwai-cho, Neyagawa-shi, Osaka 572-8572, Japan; h-tsujimoto@osaka-pct.ac.jp

[3] Kobata Sangyo Co., Ltd., 1-6-22 Kyomachibori, Nishi-ku, Osaka 550-0003, Japan; ta-nishigahana@kbts.jp (T.N.); kei_itoh2911@zeus.eonet.ne.jp (K.I.); go-kobata@kbts.jp (G.K.)

* Correspondence: nomoto@chem.osakafu-u.ac.jp; Tel.: +81-72-254-9295

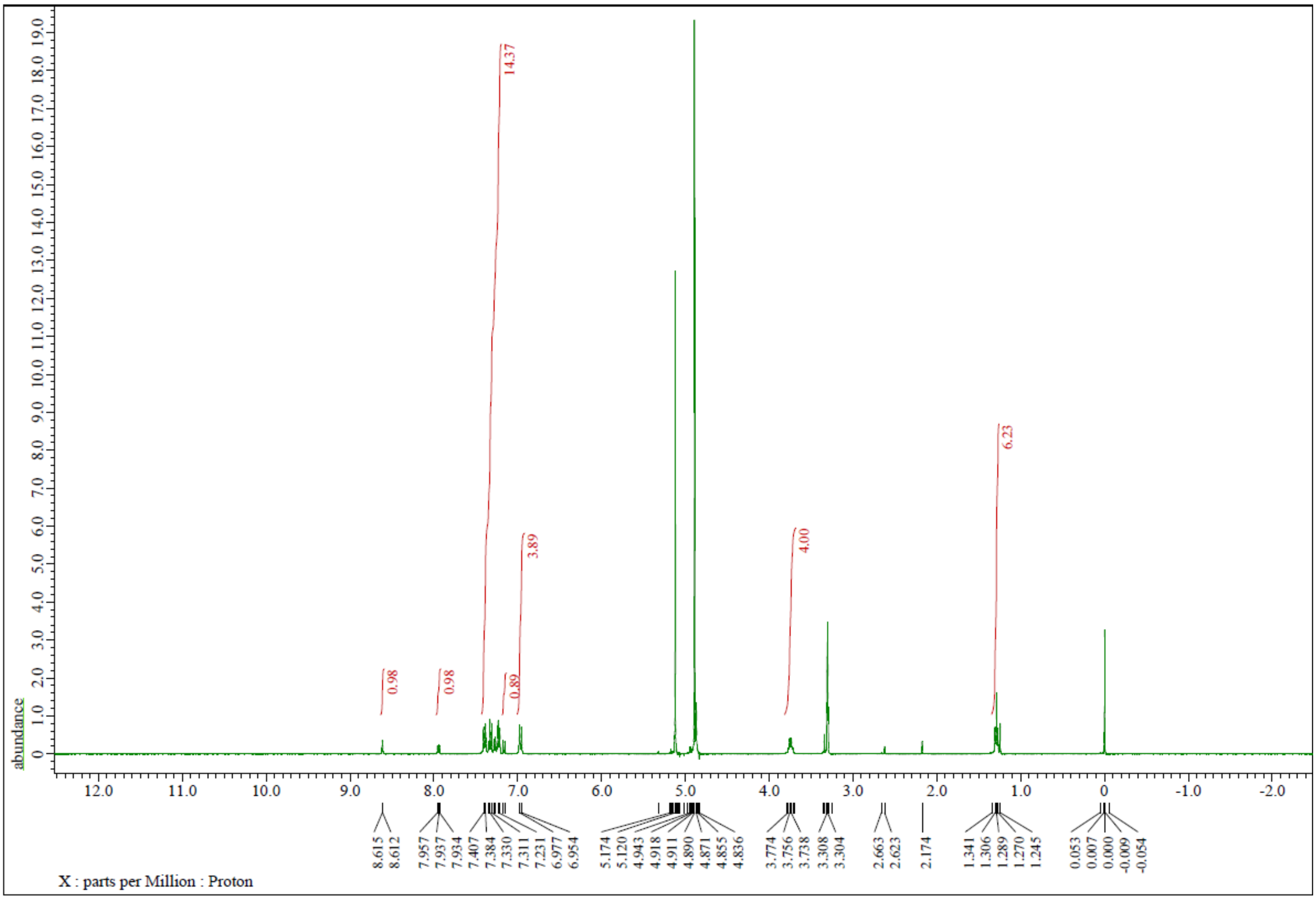

Figure S1. ^{1}H-NMR of purchased and purified Acid Blue 7 (Alphazurine A).

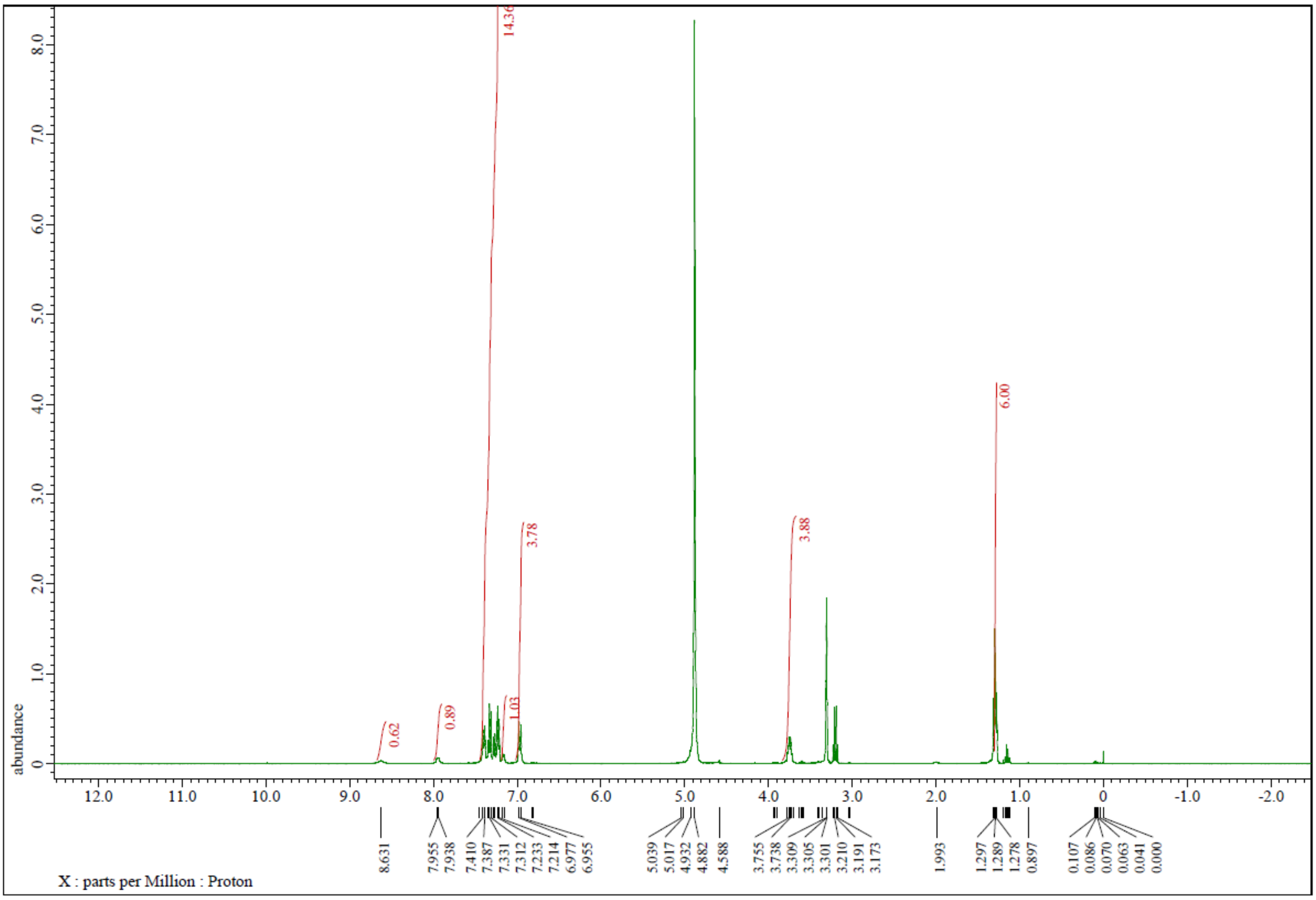

Figure S2. ^{1}H-NMR of synthesized Acid Blue 7.

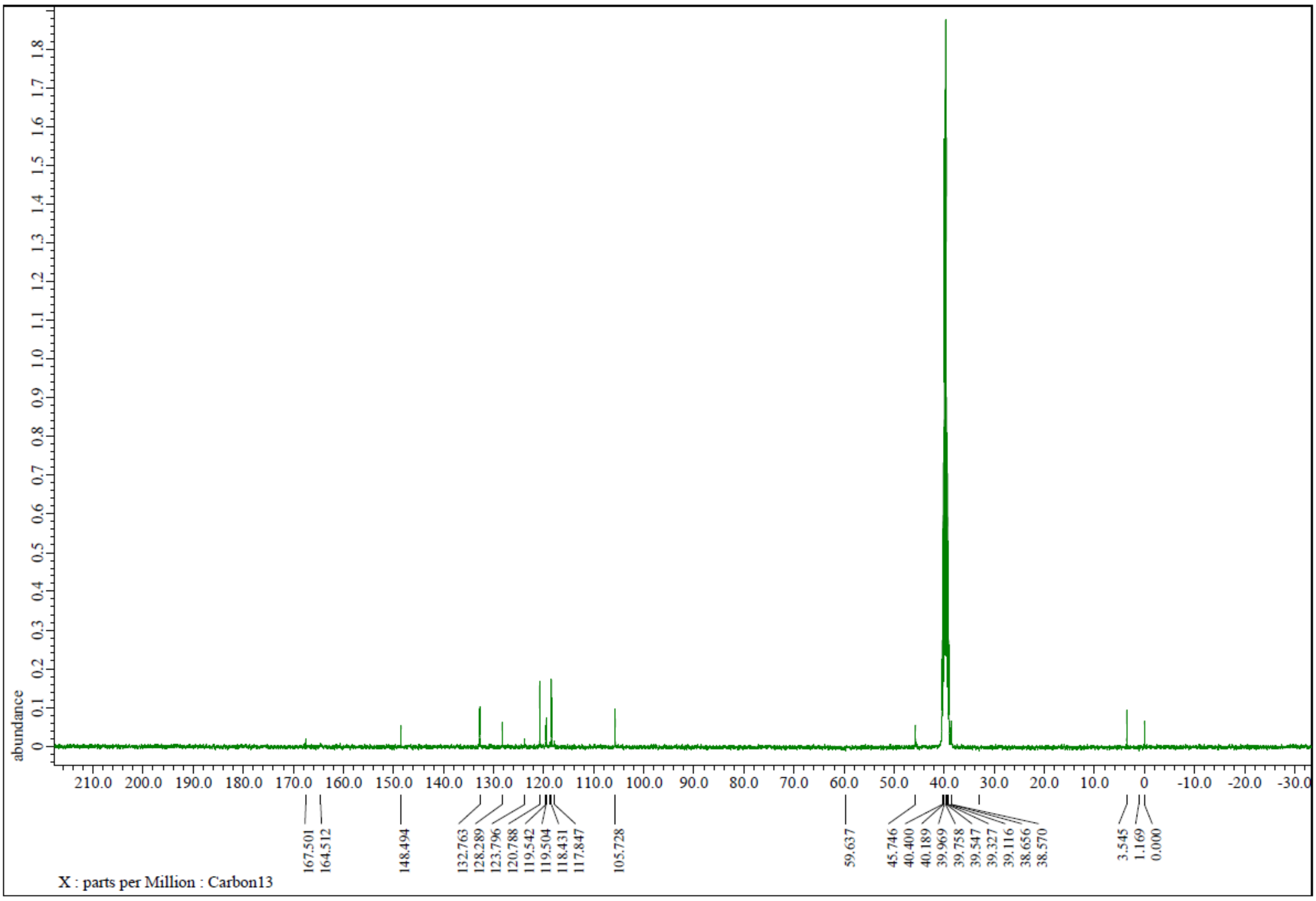

Figure S3. ^{13}C-NMR of synthesized Acid Blue 7.

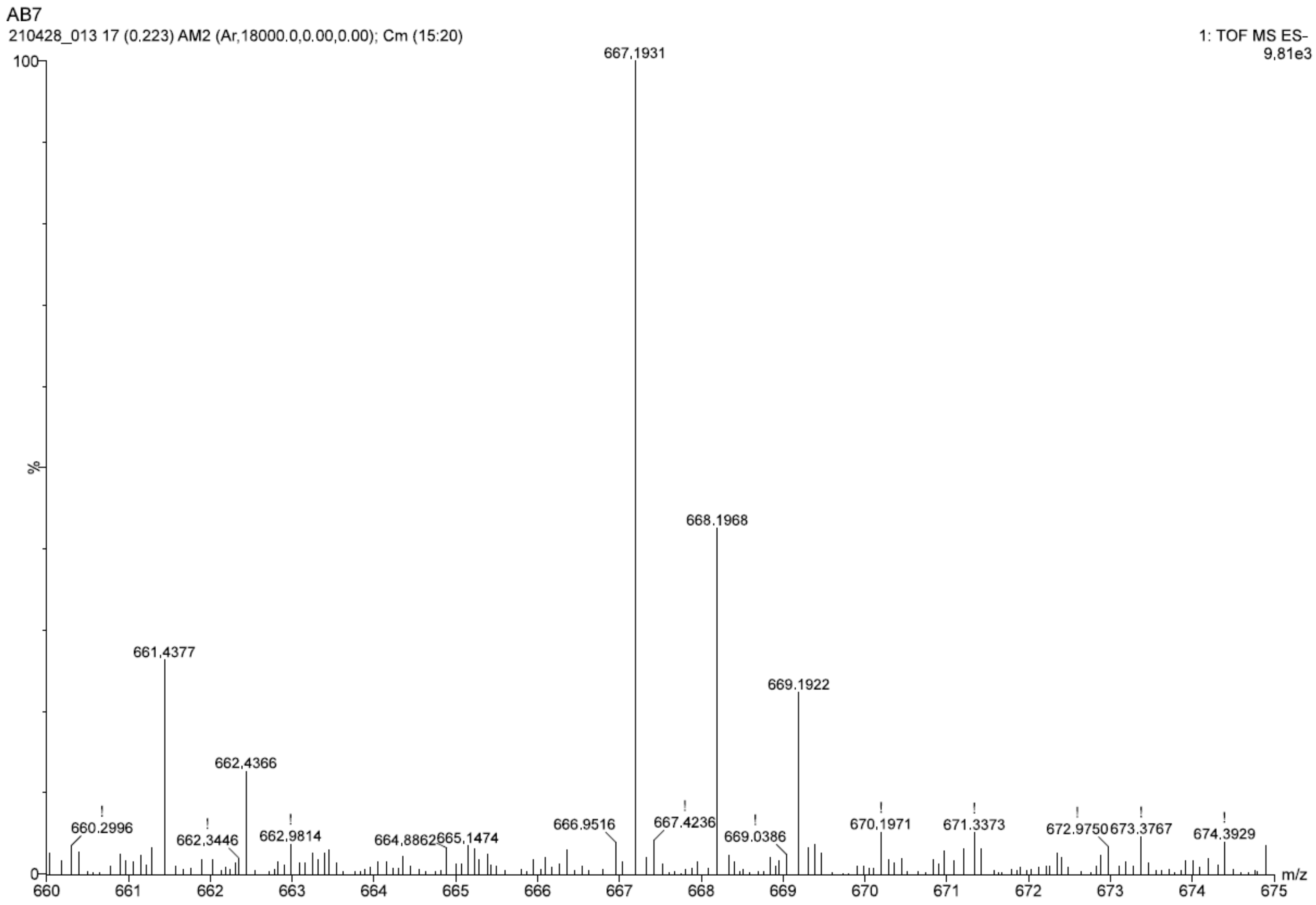

Figure S4. Mass spectra of synthesized Acid Blue 7.

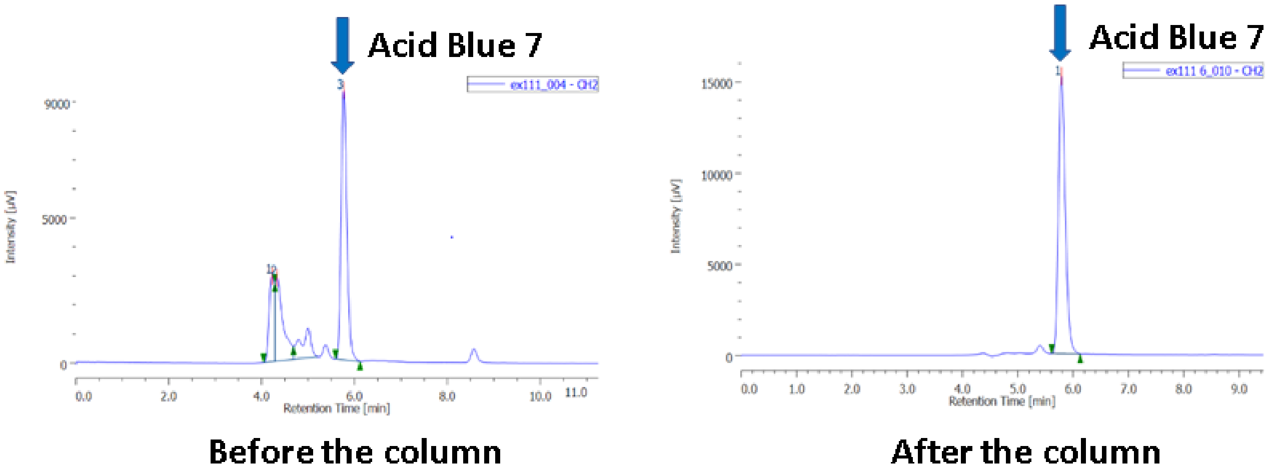

Figure S5. HPLC results of 10 gram-scale synthesis; before purification (left) and after purification (right) by column chromatography.
